# Supplementary material for: Lewis Blood-group Antigens Are Associated With Altered Susceptibility to Shigellosis
Source: Clin Infect Dis. 2020 Sep 17;72(11):e868–71. doi: 10.1093/cid/ciaa1409 (PMC8315233; doi:10.1093/cid/ciaa1409)
Supplement: ciaa1409_suppl_Supplementary_Material [file ciaa1409_suppl_supplementary_material.docx]

**Supplemental Material**

Supplementary Methods

**Flow Cytometry.**  Flow cytometry was used to measure changes in Lewis antigens and H-antigen on HT-29 cells following treatment with 2F-PAF. HT-29 cells were treated with 250μM 2F-peracetyl-fucose or vehicle as described earlier. Cells were removed from the culture dish with 0.25% trypsin-EDTA (Gibco). Following a wash, cells were stained with a fixable viability dye ZombieAqua (BioLegend) and then incubated with primary antibodies, mouse anti-Lewis B [clone 2-25LE] (Abcam), mouse anti-Lewis A [clone 7LE] (Abcam), or mouse anti-blood group H [97-I] (Invitrogen). Following incubation with primary antibodies, cells were washed and incubated with the appropriate fluorescently labeled secondary antibody, rat anti-mouse IgG1-PE (BioLegend) or rat anti-mouse IgM-APC (BioLegend). Following staining, samples were fixed with IC fixation buffer (eBioscience) and analyzed on a BD LRSFortesssa. FCS files were analyzed using the software FlowJo.


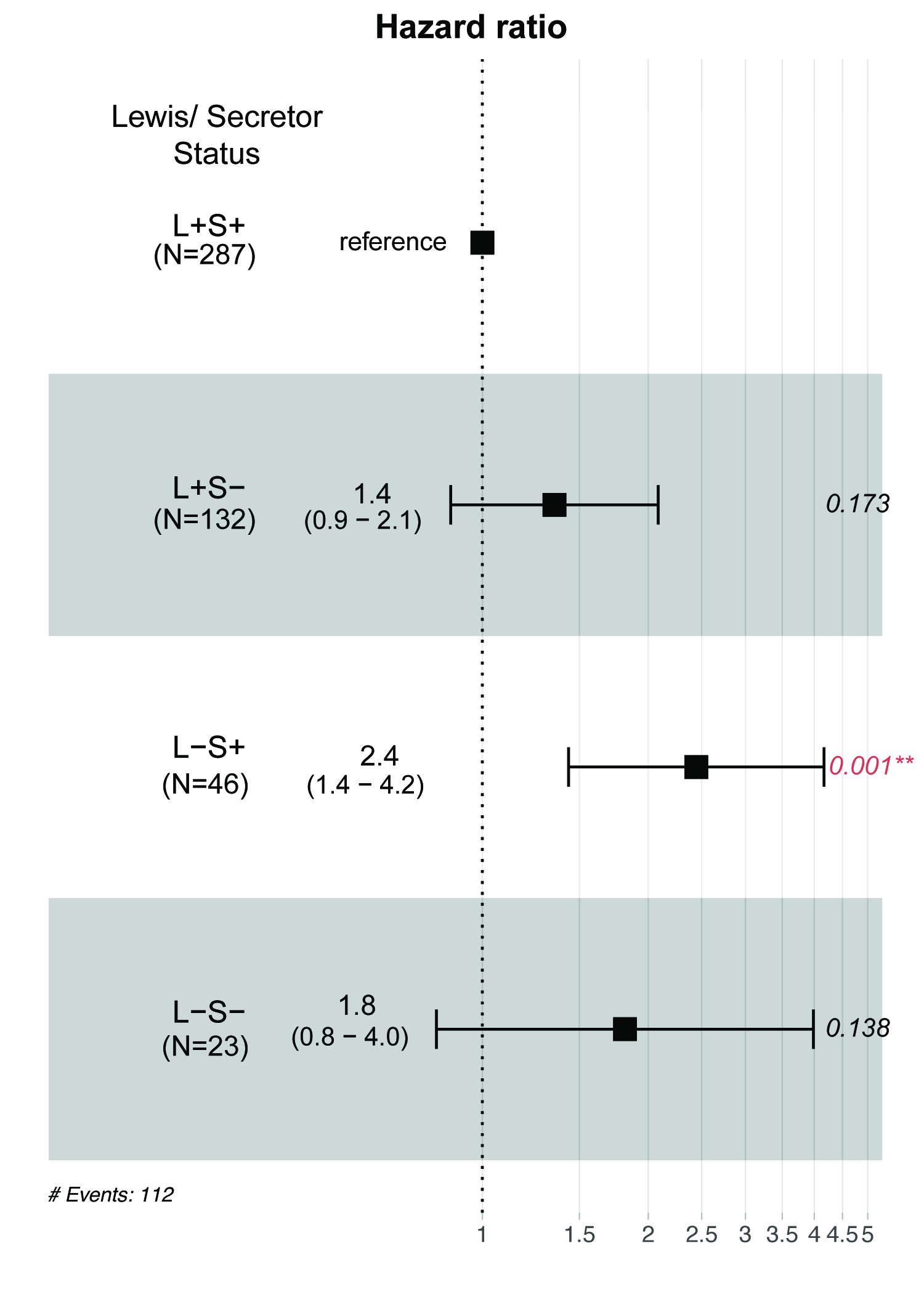


**Supplemental Figure 1: Le-/S+ infants have significantly increased risks of *Shigella* attributable diarrhea** **compared** **to Le+/Se+ infants.** Forest plot showing hazard ratios from the Cox proportional hazards model of the survival data shown in figure 1A.


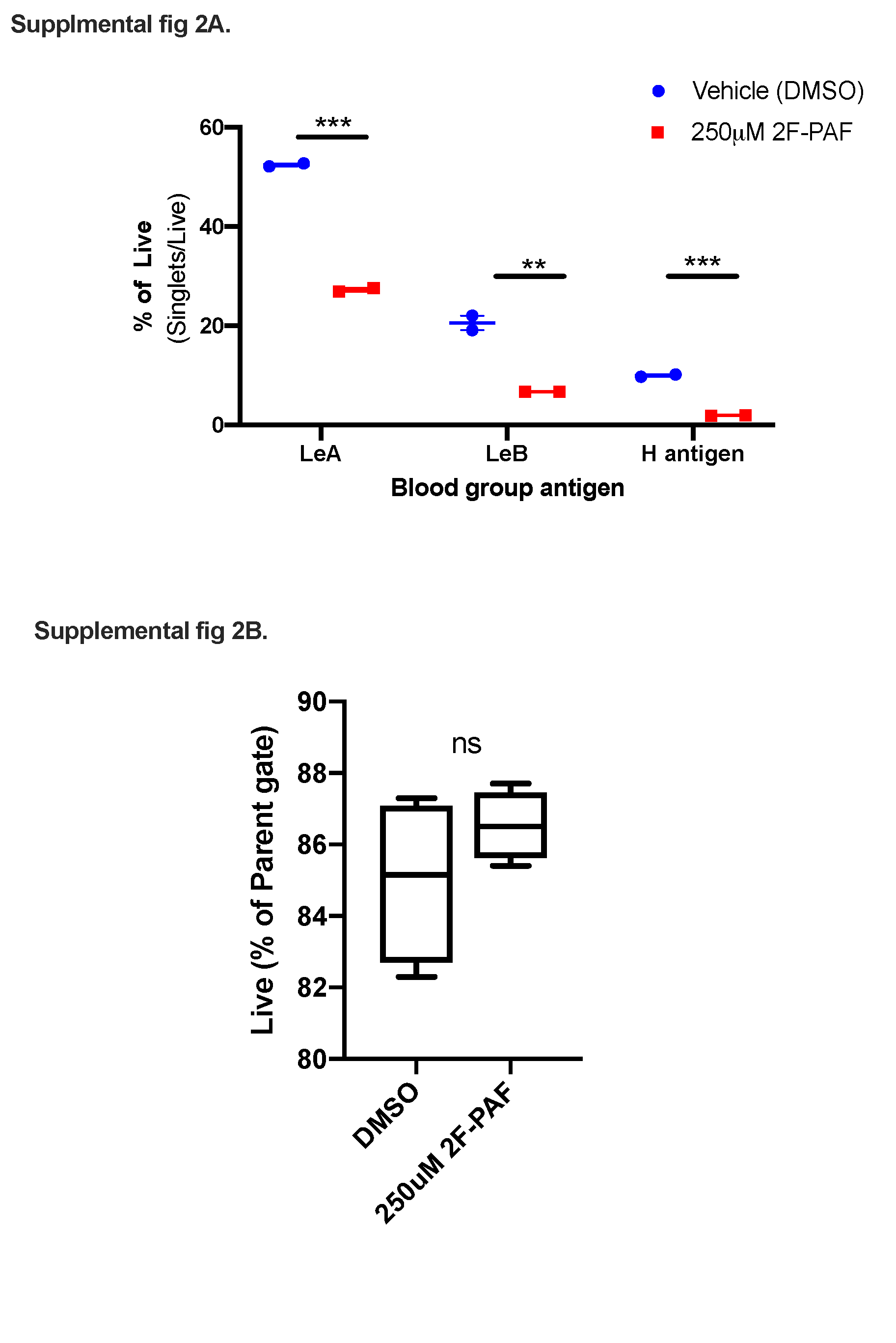


**Supplemental Figure 2: 2F-peracetyl-fucose treatment of HT-29 cells decreases Lewis A, Lewis B and H-antigen abundance.**

HT29 cells were treated with 2F-Peracetyl-Fucose (2F-PAF) or vehicle control for three days. A) 2F-PAF treatment decreased the percent of cells staining positive for Lewis A (LeA), Lewis B (LeB), and H-antigen compared to vehicle control. N=2 per group. Y-axis values represent cells staining positive for a given antigen as a percentage of parent gate (live cells). Significance was determined t- test. **, *P* < 0.01, ***, *P* < 0.001. B) 2F-PAF treatment did not significantly alter viability N=10 per group. Significance was determined by two-tailed nonparametric Mann–Whitney U test.
